# Effect of botulinum toxin type A on non-motor symptoms and quality of life in Meige syndrome

**DOI:** 10.3389/fneur.2023.1115482

**Published:** 2023-02-09

**Authors:** Heqing Zheng, Lanxiang Wu, Sheng Tian, Mingxu Liu, Qingqing Zhan, Xinping Yu, Yonggang Xie, Xianhui Zhong, Wei Wu

**Affiliations:** Department of Neurology, The Second Affiliated Hospital of Nanchang University, Nanchang, Jiangxi, China

**Keywords:** Meige syndrome, non-motor symptoms, botulinum toxin, psychiatric disturbances, quality of life

## Abstract

**Background:**

It has been shown in previous studies that botulinum toxin type A (BTX-A) can effectively relieve the motor symptoms of Meige syndrome. However, its effect on non-motor symptoms (NMS) and quality of life (QoL) has not been comprehensively studied. This study aimed to explore the effects of BTX-A on NMS and QoL and to clarify the relationship between changes in motor symptoms, NMS, and QoL after BTX-A.

**Methods:**

Seventy-five patients were recruited for the study. All patients were assessed by a series of clinical assessments before, one, and 3 months after BTX-A treatment. Dystonic symptoms, psychiatric disturbances, sleep disorders, and QoL were evaluated.

**Results:**

After 1 and 3 months of BTX-A treatment, the scores of motor symptoms, anxiety, and depression were significantly decreased (*P* < 0.05). Except for general health, the scores of the other 36-item short-form health survey QoL subitems were significantly improved after BTX-A (*P* < 0.05). After 1 month of treatment, the changes in anxiety and depression were not correlated with changes in motor symptoms (*P* > 0.05). Still, they were negatively correlated with changes in physical functioning, role-physical and mental component summary QoL (*P* < 0.05).

**Conclusions:**

BTX-A effectively improved motor symptoms, anxiety, depression, and QoL. Anxiety and depression improvement did not correlate with motor symptom changes after BTX-A, and QoL improvements were strongly associated with psychiatric disturbances.

## 1. Introduction

Meige syndrome is a rare segmental craniocervical dystonia characterized by blepharospasm and oromandibular dystonia. It can be divided into three types according to clinical manifestations: blepharospasm, blepharospasm-oromandibular dystonia, and oromandibular dystonia (1). The pathogenesis of primary Meige syndrome is unclear. It has been suggested in some studies that it is primarily related to an abnormal acetylcholine/dopamine balance. In contrast, others suggest that it may be related to reduced cortical inhibition and changes in neuronal plasticity due to environmental stimuli and genetic susceptibility (2, 3). Blepharospasm is the most common and disabling manifestation, which spreads to the muscles of the lower face, mouth, jaw, and tongue in about 50% of patients and even progresses to the trunk and limbs in a few cases (1, 4–6). Although Meige syndrome is mainly characterized by motor symptoms, it has been more frequently shown that non-motor symptoms (NMS) play an essential role in the occurrence and development of the disease and are the determinants of quality of life (QoL). In Meige syndrome, the most frequent NMS are psychiatric disturbances (e.g., anxiety, depression, and obsessive-compulsive disorder), sleep disorders, and cognition impairment (7–11).

Furthermore, it has been suggested in previous studies that NMS may be secondary to motor symptoms (12, 13). As research progresses, it has been reported by many studies that NMS is independent of motor manifestations. In some cases, it even precedes motor symptoms and may be the primary manifestation of the disease (10, 14–16). However, the relationship between motor and NMS is still a matter of debate, deserving further investigation.

Since intramuscular injection of botulinum toxin type A (BTX-A) is well tolerated and effective in determining dystonia improvement, it is currently considered the primary treatment for Meige syndrome (17, 18). The botulinum toxin is a neurotoxin produced by *Clostridium botulinum*, which can prevent neuromuscular signaling by inhibiting the release of acetylcholine from neuromuscular junctions to achieve biochemical denervation of muscle relaxation (19). However, the effects of BTX-A on NMS and QoL in Meige syndrome have been investigated in a few studies (20, 21), and they focused on only one symptom. Additionally, there is a lack of research on the association between motor symptoms, NMS, and QoL after BTX-A treatment.

In this study, we aimed to investigate the possible effects of BTX-A treatment on the non-motor burden in Meige syndrome patients, including psychiatric symptoms and sleep disorders. In addition, we aimed to evaluate the relationship between motor manifestations, NMS, and QoL before BTX-A treatment, one, and 3 months after BTX-A injection.

## 2. Materials and methods

### 2.1. Participants

Participants with Meige syndrome were consecutively recruited from the Department of Neurology, the Second Affiliated Hospital of Nanchang University, from December 2020 to November 2021. The patients were included in the experiment according to the following inclusion criteria: (1) meeting the diagnostic criteria for Meige syndrome (1), (2) the patients could fully understand the rating scale and complete it for follow-up, and (3) it was at least 3 months after the last BTX-A injection. The patients were excluded from the experiment according to the following exclusion criteria: (1) secondary Meige syndrome, (2) a history of psychosis or use of antipsychotic drugs, (3) previously diagnosed sleep disorders or use of sleeping medication, and (4) patients with severe heart disease, lung disease, liver, and kidney dysfunction, coagulation dysfunction, and hematopoietic system. The treatment with BTX-A of all patients was completed by the same experienced and qualified neurologist. The Institutional Review Board of the Second Affiliated Hospital of Nanchang University approved this study. Informed consent was obtained from all participants.

### 2.2. Clinical assessment

Demographic and clinical information was recorded for all patients, including age, gender, education, age of onset, duration of illness, history of previous illness, brain CT/MRI, and injection dose of BTX-A. All patients underwent three evaluations of motor symptoms, NMS, and QoL by the same doctor before (baseline), one, and 3 months after BTX-A treatment. The baseline evaluation was completed immediately before the new BTX-A treatment.

#### 2.2.1. Motor assessment

The Jankovic Rating Scale (22), the Blepharospasm Disability Index (BSDI) (22), and the Burke-Fahn-Marsden Dystonia Rating Scale (BFMDRS) (23) were used to evaluate the motor symptoms of Meige syndrome patients. The JRS was given to assess the severity of blepharospasm, including severity and frequency. The BSDI for assessing blepharospasm disability included six components: driving a vehicle, reading, watching TV, shopping, walking, and doing everyday activities. The BFMDRS has two parts: the movement subscale and the disability subscale, which are used to assess the severity of the disease and the disability of daily activities individually.

#### 2.2.2. Non-motor assessment

The severity of anxiety and depression symptoms were evaluated by the Hamilton Anxiety Rating Scale (HAMA) (24) and the 17-item Hamilton Depression Rating Scale (HAMD) (25), respectively. A score of HAMA more than 6 indicates anxiety and HAMD more than 7 indicates depression. The sleep quality was measured using the Pittsburgh sleep quality index (PSQI) (26). A score of 6 or more is considered to have sleep disorder. The severity of insomnia was assessed using the Insomnia Severity Index (ISI) (27). A score of ISI >7 indicates insomnia. The excessive daytime sleepiness was assessed using the Epworth Sleepiness Scale (ESS) (28). If the patient reached an ESS index higher than or equal to 10, the patient was considered to have excessive daytime sleepiness.

#### 2.2.3. QoL assessment

QoL was measured with a widely used questionnaire of a 36-item short-form health survey (29) (SF-36), consisting of eight domains of physical and mental health: physical functioning (PF), role-physical (RP), bodily pain (BP), general health (GH), Vitality (VT), social functioning (SF), role-emotional (RE), and mental health (MH). The physical component summary (PCS) included PF, RF, BP, GH, and the mental component summary (MCS) included VT, SF, RE, and MH.

### 2.3. Statistical analysis

All statistical analyses were performed using BMI SPSS Statistics 26.0 (SPSS, Inc., Chicago, IL). Two-sided tests (*P* < 0.05) were considered statistically significant. The Kolmogorov–Smirnov test was used to examine the normality of continuous variables. Data with normal distribution were expressed as mean ± standard deviation (SD). Measurement data with a non-normal distribution were expressed as the median and interquartile range (IQR). The Wilcoxon signed rank sum test was used to compare groups before treatment and one and 3 months after treatment. Count data were expressed as relative number constituent ratio (%) or rate (%), and comparison between groups was analyzed using the chi-square test or Fisher's exact probability method. The comparison between the subtypes was carried out by independent-sample *t*-test or non-parametric test. Spearman's rank correlation coefficient was used to analyze the relationship between NMS, motor symptoms, and QoL.

## 3. Results

### 3.1. Demographic information and clinical characteristics

Data from 75 patients with Meige syndrome were collected in our study. The demographic information and clinical characteristics of these patients are shown in Table 1. Their average age was 60.12 ± 9.82 years, and 59 patients (78.67%) were female. The mean age of onset was 56.03 ± 9.61 years, the median duration of the disease was 2.5 years (IQR: 0.75–5), and 43 cases (57.33%) had sensory tricks. Thirty-seven (49.33%) patients were treated with BTX-A for the first time. There were 58 (77.33%) patients with blepharospasm, and 17 (22.67%) patients with blepharospasm combined with oromandibular dystonia.

**Table 1:** Demographic information and clinical characteristics in Meige.

**Demographic and clinical data**	**Meige patients**
Age (years), mean ± SD	60.12 ± 9.82
Sex (female), *n* (%)	59 (78.67%)
Age onset (years), mean ± SD	56.03 ± 9.61
Disease duration (years)	2.5 (0.75, 5)
Sensory tricks, *n* (%)	43 (57.33%)
First-time injection, *n* (%)	37 (49.33%)
Subtypes, *n* (%)	
Blepharospasm	58 (77.33%)
Blepharospasm-oromandibular dystonia	17 (22.67%)
HAMA	6 (2, 11)
HAMD	8 (4, 11)
PSQI	5 (2, 9)
ESS	5 (5, 7)
ISI	3 (1, 11)
Anxiety, *n* (%)	32 (42.67%)
Depression, *n* (%)	40 (53.33%)

Values are medians (IQR) unless otherwise indicated. HAMA, Hamilton Anxiety Rating Scale; HAMD, Hamilton Depression Rating Scale; PSQI, Pittsburgh Sleep Quality Index; ESS, Epworth Sleepiness Scale; ISI, Insomnia Severity Index.

### 3.2. Effects of BTX-A on motor symptoms

As shown in Table 2, the JRS total score, JRS frequency score, JRS severity score, BSDI score, BFMDRS-M, and BFMDRS-D scores of Meige syndrome patients were lower after one and 3 months of BTX-A treatment (*P* < 0.05). However, there was no significance in the neck subscale of BFMDRS-M after treatment (*P* > 0.05).

**Table 2:** Changes of motor symptoms after BTX-A treatment in patients with Meige syndrome.

**Variable**	**Baseline**	**1 month after BTX-A**		**3 months after BTX-A**	
	**scores**	**scores**	***P***	**scores**	***P***
JRS total score (range, 0–8)	7 (6, 7)	2 (0, 2)	< 0.001	2 (0, 4)	< 0.001
JRS frequency score (range, 0–4)	4 (3, 4)	1 (0, 1)	< 0.001	1 (0, 2)	< 0.001
JRS severity score (range, 0–4)	3 (3, 3)	1 (0, 1)	< 0.001	1 (0, 1)	< 0.001
BSDI (range, 0–24)	15 (12, 17)	0 (0, 5)	< 0.001	0 (0, 5)	< 0.001
BFMDRS-M (range, 0–40)	8 (8, 8)	1.5 (0, 3)	< 0.001	3 (0, 4.5)	< 0.001
Eye (range, 0–8)	8 (8, 8)	1.5 (0, 3)	< 0.001	0 (0, 0)	< 0.001
Mouth (range, 0–8)	0 (0, 0)	0 (0, 0)	< 0.001	0 (0, 0)	0.001
Neck (range, 0–8)	0 (0, 0)	0 (0, 0)	0.068	0 (0, 0)	0.068
Speech and swallowing (range, 0–16)	0 (0, 0)	0 (0, 0)	0.017	0 (0, 0)	0.016
BFMDRS-D (range, 0–30)	3 (2, 4)	0 (0, 1)	< 0.001	1 (0, 2)	< 0.001

Values are medians (IQR) unless otherwise indicated. JRS, Jankovic Rating Scale; BSDI, Blepharospasm Disability Index; BFMDRS-M, Burke-Fahn-Marsden Dystonia Rating Scale movement subscales; BFMDRS-D, Burke-Fahn-Marsden Dystonia Rating Scale disability subscales.

The effects of BTX-A on motor symptoms of Meige syndrome subtypes are shown in **Table 4**. The JRS total score, BSDI score, BFMDRS-M, and BFMDRS-D scores of two subtypes were lower after 1 month of BTX-A treatment (*P* < 0.05). Compared with the blepharospasm, the blepharospasm combined with oromandibular dystonia had higher BFMDRS-M scores before and 1 month after BTX-A treatment (*P* < 0.05).

### 3.3. Effects of BTX-A on non-motor symptoms

**Figure 1:**
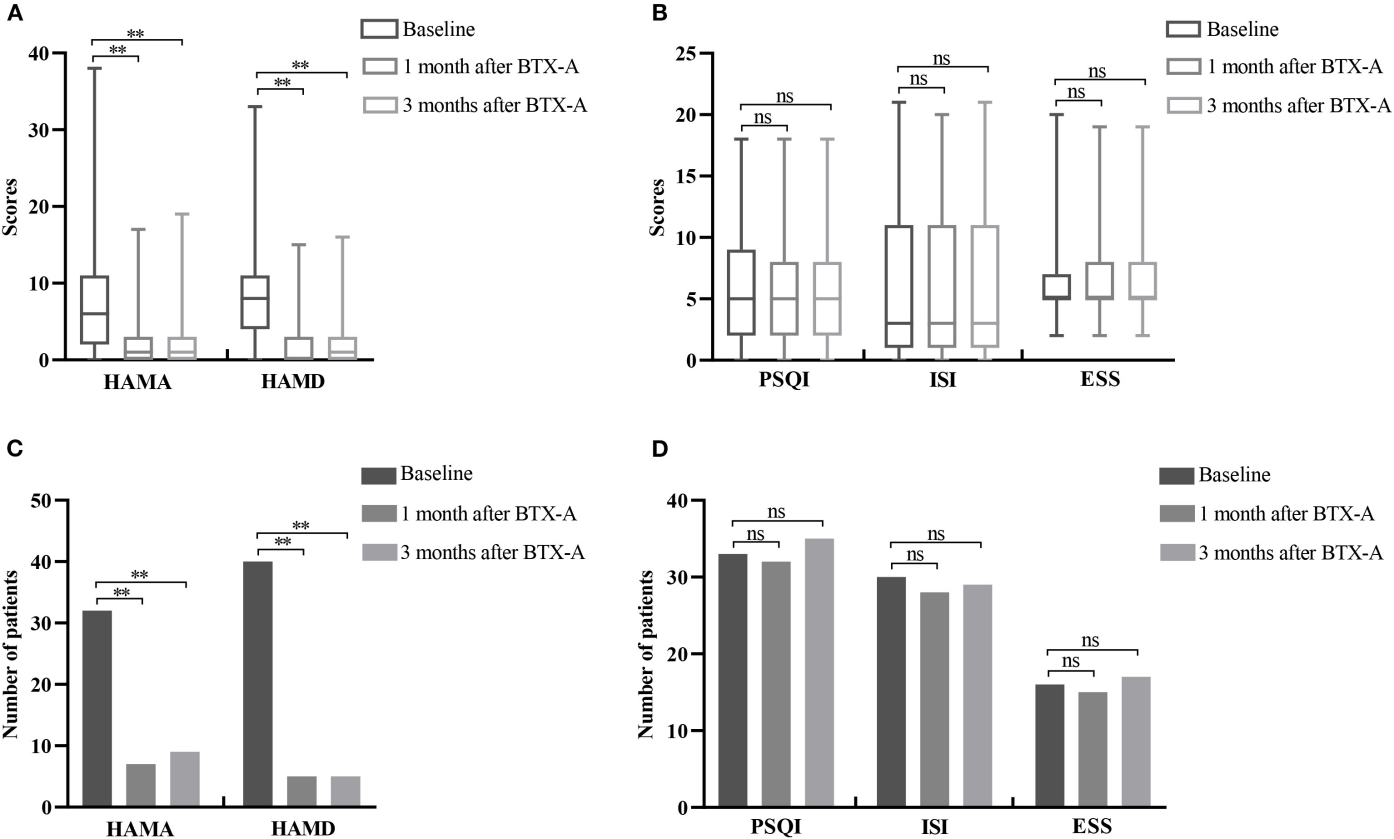
Changes of non-motor symptoms after BTX-A treatment in patients with Meige syndrome. **(A)** HAMA and HAMD scores at baseline and 1, 3 months after BTX-A treatment. **(B)** PSQI, ISI, and ESS scores at baseline and 1, 3 months after BTX-A treatment. **(C)** Number of patients with depression and anxiety at baseline and 1, 3 months after BTX-A treatment. **(D)** Number of sleep disorders at baseline and 1, 3 months after BTX-A treatment. HAMA, Hamilton Anxiety Rating Scale; HAMD, Hamilton Depression Rating Scale; PSQI, Pittsburgh Sleep Quality Index; ESS, Epworth Sleepiness Scale; ISI, Insomnia Severity Index. ^**^P < 0.001; ^ns^*P* > 0.05.

The HAMA and HAMD scores of Meige syndrome patients decreased at 1 and 3 months after BTX-A treatment (*P* < 0.05) (Figure 1A). There were 32 (42.67%) patients with anxiety before treatment (Table 1), seven patients at 1 month, and nine patients at 3 months after treatment (Figure 1C). There were 40 (53.33%) patients with depression before treatment (Table 1) and five patients 1 and 3 months after treatment (Figure 1C). There was a statistical difference in reducing the number of patients with anxiety and depression after treatment. However, there was no significance in PSQI, ISI, and ESS scores after BTX-A (*P* > 0.05) (Figure 1B), nor in the number of sleep disturbances (*P* > 0.05) (Figure 1D).

The HAMA and HAMD scores of two subtypes were lower after BTX-A treatment. There was no significance in HAMA and HAMD scores of the two subtypes 1 month after BTX-A treatment (*P* > 0.05) (**Table 4**).

### 3.4. Effects of BTX-A on QoL

In Table 3, after one and 3 months of BTX-A treatment, the scores of PF, RP, BP, VT, RE, and MH in the SF-36 scale of patients with Meige syndrome improved (the higher the score, the higher the QoL) (*P* < 0.05), but there was no significant change in GH (*P* > 0.05).

**Table 3:** Changes of quality of life after BTX-A treatment in patients with Meige syndrome.

**SF-36**	**Baseline**	**1 month after BTX-A**		**3 months after BTX-A**	
	**scores**	**scores**	***P***	**scores**	***P***
Physical functioning	60 (45, 75)	100 (85, 100)	< 0.001	100 (80, 100)	< 0.001
Role physical	25 (0, 50)	100 (50, 100)	< 0.001	100 (50, 100)	< 0.001
Bodily pain	90 (90, 90)	90 (90, 90)	< 0.001	90 (90, 90)	0.001
General health	52 (45, 57)	52 (52, 55)	0.587	52 (52, 57)	0.475
Vitality	75 (60, 85)	90 (90, 95)	< 0.001	90 (85, 95)	< 0.001
Social functioning	77.78 (66.67, 88.89)	100 (100, 100)	< 0.001	100 (88.89, 100)	< 0.001
Role emotional	100 (100, 100)	100 (100, 100)	< 0.001	100 (100, 100)	< 0.001
Mental health	72 (56, 84)	100 (100, 100)	< 0.001	92 (88, 92)	< 0.001

Values are medians (IQR) unless otherwise indicated. SF-36, 36-item short-form health survey.

In Table 4, there was no significance in GH of the blepharospasm, BP, and GH of the blepharospasm combined with oromandibular dystonia 1 month after BTX-A (*P* > 0.05). The PF, BP, and VT scores of blepharospasm improved more after 1 month of BTX-A treatment (*P* < 0.05).

**Table 4:** Results of BTX-A injection for each subtype of Meige syndrome.

**Variable**	**Blepharospasm (58)**	**Blepharospasm-oromandibular dystonia (17)**	***P***
Age (years), mean ± SD	58.26 ± 9.84	66.47 ± 6.77	< 0.001
Sex (female), *n* (%)	43 (74.14%)	16 (94.12%)	0.152
Age onset (years), mean ± SD	54.91 ± 9.82	59.82 ± 7.99	0.064
Disease duration (years)	1.75 (0.50, 4.25)	4.00 (2.75, 9.5)	0.002
No. of BTX-A injection, mean ± SD	19.40 ± 4.05	30.53 ± 9.74	< 0.001
No. of injected muscles, mean ± SD	8.76 ± 3.32	14.76 ± 4.44	< 0.001
Dose of BTX-A injection (U), mean ± SD	47.50 ± 11.49	77.16 ± 28.83	0.001
**Baseline**			
JRS total score (range, 0–8)	7 (6, 7)	7 (7, 7)	0.488
BSDI (range, 0–4)	15 (12, 18)	15 (11, 15)	0.271
BFMDRS-M (range, 0–40)	8 (7.5, 8)	14 (10, 18.5)	< 0.001
BFMDRS-D (range, 0–30)	3 (2, 4)	3 (2, 5.5)	0.587
HAMA	6 (2, 10)	6 (2.5, 17)	0.585
HAMD	8 (4,11)	8 (4, 12.50)	0.616
**SF-36**			
Physical functioning	60 (45, 75)	70 (47.50, 82.50)	0.509
Role physical	25 (0, 50)	25 (0, 50)	0.538
Bodily pain	90 (90, 90)	90 (82, 90)	0.199
General health	52 (46.5, 57)	45 (41, 53.50)	0.054
Vitality	75 (60, 85)	75 (62.50, 85)	0.858
Social functioning	77.78 (63.89, 88.89)	66.67 (55.56, 94.45)	0.898
Role emotional	83.34 (33.3, 100)	100 (16.67, 100)	0.870
Mental health	72 (60, 84)	76 (52, 84)	0.476
**1 month after BTX-A**			
JRS total score (range, 0–8)	0 (0, 2)	2 (2, 3)	0.009
BSDI (range, 0–4)	15 (12, 18)	1 (0, 5)	0.753
BFMDRS-M (range, 0–40)	0 (0, 3)	4.5 (1.5, 6.75)	< 0.001
BFMDRS-D (range, 0–30)	0 (0, 1)	1 (0, 1)	0.056
HAMA	0.5 (0, 3)	1 (0, 4)	0.284
HAMD	0 (0, 3)	2 (0, 3.5)	0.283
**SF-36**			
Physical functioning	100 (90, 100)	85 (80, 100)	0.006
Role physical	100 (50, 100)	100 (50, 100)	0.171
Bodily pain	90 (90, 90.5)	90 (87, 90)	0.006
General health	52 (52, 52)	52 (50, 56)	0.708
Vitality	90 (90, 95)	90 (75, 90)	0.007
Social functioning	100 (100, 100)	100 (83.34, 100)	0.100
Role emotional	100 (100, 100)	100 (100, 100)	0.334
Mental health	92 (92, 92)	92 (68, 92)	0.321

Values are medians (IQR) unless otherwise indicated. JRS, Jankovic Rating Scale; BSDI, Blepharospasm Disability Index; BFMDRS-M, Burke-Fahn-Marsden Dystonia Rating Scale movement subscales; BFMDRS-D, Burke-Fahn-Marsden Dystonia Rating Scale disability subscales; HAMA, Hamilton Anxiety Rating Scale; HAMD, Hamilton Depression Rating Scale; SF-36, 36-item short-form health survey.

### 3.5. Relationship between motor, non-motor, and QoL

#### 3.5.1. Relationship between motor and non-motor

Spearman's rank correlation coefficient was used to examine the correlation between the severity of NMS (HAMA, HAMD, PSQI, ISI, and ESS) and the severity of motor symptoms (JRS, BSDI) in patients with Meige's syndrome, and the results showed no correlation (*P* > 0.05). After correcting for HAMA and HAMD factors using regression analysis, the PSQI, ISI, and ESS scores were still not related to the severity of motor symptoms (*P* > 0.05).

We discovered that Meige syndrome patients showed improvements in motor symptoms, anxiety, and depression after BTX-A treatment. We analyzed their changes after 1 month of BTX-A treatment using Spearman's correlation analysis to clarify their relationship further. It was shown that the improvement in anxiety and depression after BTX-A treatment was unrelated to the improvement in motor symptoms (*P* > 0.05).

#### 3.5.2. Effects of motor and non-motor symptoms on QoL

We used Spearman's rank correlation coefficient to investigate the impact of motor and NMS on QoL in patients with Meige syndrome. We observed that JRS and BSDI scores were negatively correlated with PF and RP scores (*P* < 0.05), with JRS scores also negatively associated with BP and SF scores (*P* < 0.05). HAMA and HAMD scores were negatively correlated with PF, GH, and MCS QoL subscales (VT, SF, RE, and MH) scores (*P* < 0.05).

We investigated the effects of motor and psychiatric symptoms on QoL BTX-A-induced changes in Meige syndrome patients using Spearman's correlation analysis. After 1 month of BTX-A treatment, the changes in JRS and BSDI were negatively correlated with the changes in PF and RP scores (*P* < 0.05). Changes in HAMA and HAMD scores were negatively correlated with changes in RF, RP, and MCS QoL subscales (VT, SF, RE, and MH) scores (*P* < 0.05) (Table 5).

**Table 5:** Effects of motor and non-motor symptoms on quality of life BTX-A-induced changes.

**SF-36**	Δ**JRS**		Δ**BSDI**		Δ**HAMA**		Δ**HAMD**	
	***r***	***P***	***r***	***P***	***r***	***P***	***r***	***P***
ΔPF	−0.363	0.001	−0.370	0.001	−0.305	0.008	−0.534	< 0.001
ΔRP	−0.405	< 0.001	−0.398	< 0.001	−0.275	0.017	−0.297	0.010
ΔBP	−0.139	0.233	−0.130	0.265	−0.206	0.076	−0.095	0.415
ΔGH	–	–	–	–	–	–	–	–
ΔVT	−0.150	0.200	−0.120	0.307	−0.463	< 0.001	−0.500	< 0.001
ΔSF	−0.134	0.253	−0.075	0.522	−0.282	0.014	−0.501	< 0.001
ΔRE	−0.084	0.472	−0.148	0.206	−0.299	0.009	−0.364	0.001
ΔMH	−0.051	0.664	−0.092	0.434	−0.485	< 0.001	−0.654	< 0.001

SF-36, 36-item short-form health survey; JRS, Jankovic Rating Scale; BSDI, Blepharospasm Disability Index; HAMA, Hamilton Anxiety Rating Scale; HAMD, Hamilton Depression Rating Scale; PF, physical functioning; RP, role-physical; BP, bodily pain; GH, general health; VT, vitality; SF, social functioning; RE, role-emotional; MH, mental health.

## 4. Discussion

This study was the first to focus on the effects of BTX-A on NMS and QoL in Meige syndrome patients. From our findings, motor symptoms, anxiety, depression, and QoL were effectively improved by BTX-A, but no significant changes in sleep were observed. Anxiety and depression improvement did not correlate with motor symptom changes after the BTX-A injection. Psychiatric disturbance changes were associated with improvements in the mental component of QoL subscales after BTX-A treatment.

We found no significant correlation between the severity of anxiety and depression and the severity of motor symptoms in patients with Meige syndrome. These results were consistent with many previous studies (15, 16, 30, 31), suggesting that NMS may be the original sign of Meige syndrome. We also observed that BTX-A could significantly improve the anxiety and depression symptoms in Meige syndrome patients. Dong et al. (20) and Yin et al. (21) also found marked differences in anxiety and depressive symptoms in patients with blepharospasm after BTX-A treatment. However, both studies suffered from a lack of assessment of motor symptoms before and after treatment, and, therefore, could not determine whether changes in psychiatric symptoms correlated with improvements in motor symptoms. Costanzo et al. (30) found that motor symptoms, anxiety, and depression were significantly improved in focal dystonia patients after BTX-A treatment. Still, variations in anxiety and depression were not linked to improvements in motor symptoms, following the results of our findings. We also found that there was no correlation between the subtypes of Meige syndrome and the improvement of anxiety and depression after BTX-A treatment. Therefore, in clinical treatment, we should pay attention to motor symptoms and assess the emotional state of patients. We should evaluate whether to treat the patients when Meige syndrome patients have a combination of anxiety and depression.

The mechanisms underlying the combination of anxiety and depression in Meige syndrome are unclear. Meige syndrome is now mostly considered a neural network disorder, and the connections between the basal ganglia and supplementary motor areas, as well as the sensorimotor cortex, supplementary motor areas, premotor cortex, precuneus, and parietal cortex, are changed. This abnormal connection may underlie the onset of NMS (32, 33). Notably, the prefrontal-limbic-striatal loop, which connects the prefrontal cortex and orbitofrontal cortex with ventral striatal areas, the ventral pallidum, and the medial thalamus, may underlie the development of psychiatric disorders. Functional magnetic resonance imaging studies indicated that BTX-A treatment could improve the balance of sensorimotor network connections in patients with Meige syndrome (34). This suggests that improvements in psychiatric symptoms may be relevant to rebalancing connections between motor and non-motor areas of the brain network in patients with Meige syndrome. It has been suggested in other studies that depressive symptoms can be improved by injecting BTX-A between the eyebrows (35–39). Also, there may be the following mechanisms: achieving mood-lifting effects through facial and social feedback (40), up-regulating brain-derived neurotrophic factor (BNDF) and monoamines (5-hydroxytryptamine or norepinephrine) levels (41), inhibiting neuroinflammation (42–44) and modifying the insular cortex (40, 45). Considering the above, we speculate that BTX-A may improve psychiatric symptoms in patients with Meige syndrome concerning the improved connectivity of brain regions and antidepressant effects alone. The mechanism remains to be further investigated in the future.

It was suggested by the results of this study that sleep disturbance in Meige syndrome was not related to the severity of motor symptoms. Similarly, it was found in previous studies that the quality of sleep in blepharospasm was not associated with the severity of the spasm. The incidence of sleep disorders in dystonia was higher than in ordinary people (8, 46, 47). It was suggested by these findings that sleep disorders may be the primary manifestation of the disease. Changes in neurotransmitter pathways or neural circuits common to motor symptoms may be the basis of sleep disorders, in which the mesencephalic-limbic dopamine pathway may play an important role (48). However, unlike motor and psychiatric symptoms, there was no obvious difference in sleep quality, insomnia, or daytime sleepiness after BTX-A treatment. Sleep disorders are ongoing or long-term processes influenced by various factors, including environmental, occupational, physiological, medical, and psychiatric disorders. As a result, if patients have comorbid sleep disorders, it is vital to take independent treatment measures to improve their sleep quality. In addition, short-term study results may not be reliable, and future longitudinal studies are needed to provide new light on treating sleep disorders in Meige syndrome with BTX-A.

After treatment with BTX-A, the QoL in Meige's syndrome patients improved notably in our study. Yoshida et al. (49) evaluated the Oromandibular Dystonia Rating Scale in 408 patients with oromandibular dystonia before and after botulinum toxin treatment. At a follow-up of over 12 months, the overall quality of life of the patients improved significantly after treatment. Ochudlo et al. (50) assessed the SF-36 scale in 33 patients with blepharospasm at follow-up after BTX-A treatment and found significant improvements in all subscales except body pain. We found that the PF, BP, and VT in blepharospasm improved more after 1 month of BTX-A. It has been reported that the post-treatment health-related quality of life are significantly different depending on the subtype of dystonia (49, 51). Therefore, the early recognition and treatment of motor symptoms of Meige syndrome are important to improve the quality of life of patients.

The QoL of people with Meige syndrome is affected not only by motor symptoms but also by NMS. It was demonstrated in our study that the PF and RP subscales of the SF-36 in patients were mainly influenced by motor symptoms. At the same time, PF, RP, GH, and MCS were affected by psychiatric disorders. Moreover, after BTX-A treatment, changes in PF and RP were associated with improvements in motor and psychiatric symptoms, and changes in the MCS QoL subscales were also related to improvements in psychiatric disorders. It was shown by these results that NMS seems to be more closely associated with QoL than motor symptoms. Recently, a large, international, multicentre cohort study of patients with idiopathic dystonia also showed that QoL was closely connected to physical and psychological characteristics (52). Therefore, we should pay more attention to whether the patient has NMS and provide intervention if necessary to improve their QoL in our clinical treatment. However, there is a lack of research regarding the impact of motor and NMS on QoL in Meige syndrome patients after BTX-A treatment. Such a direction could be explored in the future.

There are some limitations to this study. First, the study period was only from pre-treatment to 3 months post-treatment, and it was impossible to observe changes beyond 3 months of BTX-A treatment. However, BTX-A may have a long-term effect on NMS, and additional studies with prolonged follow-ups are necessary. Second, repeat bias in clinical scales cannot be completely ruled out. Finally, this study did not involve patients with oromandibular dystonia. Therefore, our study was insufficient to comprehensively analyze the impact of all subtypes of Meige syndrome on the effect of BTX-A and post-treatment health-related QoL. Further research needs to expand the sample size in the future.

## 5. Conclusions

In summary, it was demonstrated in our study that BTX-A was effective in improving motor symptoms, anxiety, depression, and QoL in patients with Meige syndrome but made no apparent alteration to sleep. However, there was no correlation between psychiatric and motor symptom changes after BTX-A treatment, suggesting that BTX-A has an independent antianxiety and depression mechanism. QoL was closely related to psychiatric and physical features in Meige syndrome patients. Therefore, during the clinical consultation and treatment process, clinicians should focus on motor symptoms, pay more attention to whether patients have combined anxiety, depression, and sleep problems, and provide interventions when necessary to improve their QoL.

## Data availability statement

The original contributions presented in the study are included in the article/supplementary material, further inquiries can be directed to the corresponding author.

## Ethics statement

The studies involving human participants were reviewed and approved by the Institutional Review Board of the Second Affiliated Hospital of Nanchang University. The patients/participants provided their written informed consent to participate in this study.

## Author contributions

HZ contributed to the conception of the work, data acquisition, statistical analysis, and writing of the first draft. LW contributed to the design and organization of the work, manuscript review, and critique. ST, ML, QZ, XY, YX, and XZ contributed to the data acquisition. WW contributed to the manuscript review and critique. All authors contributed to the article and approved the submitted version.
